# Food insecurity is associated with the sleep quality and quantity in adults: a systematic review and meta-analysis

**DOI:** 10.1017/S1368980022002488

**Published:** 2023-04

**Authors:** Seyadeh Narges Mazloomi, Sepide Talebi, Maryam Kazemi, Seyed Mojtaba Ghoreishy, Seyedeh Parisa Moosavian, Parsa Amirian, Hamed Mohammadi, Saeedeh Nouri-Majd, Wolfgang Marx, Mohammad Ali Hojjati Kermani, Sajjad Moradi

**Affiliations:** 1 The Health of Plant and Livestock Products Research Center, Mazandaran University of Medical Sciences, Sari, Iran; 2 Food and Drug Administration, Mazandaran University of Medical Sciences, Sari, Iran; 3 Department of Clinical Nutrition, School of Nutritional Sciences and Dietetics, Tehran University of Medical Sciences, Tehran, Iran; 4 Hilda and J. Lester Gabrilove Division of Endocrinology, Diabetes, and Bone Disease, Department of Medicine, Icahn School of Medicine at Mount Sinai, New York, NY, USA; 5 Department of Community Nutrition, Vice-Chancellery for Health, Shiraz University of Medical Sciences, Shiraz, Iran; 6 General Practitioner, Kermanshah University of Medical Sciences (KUMS), Kermanshah, Iran; 7 Department of Community Nutrition, School of Nutritional Sciences and Dietetics, Tehran University of Medical Sciences, Tehran, Iran; 8 Deakin University, IMPACT – the Institute for Mental and Physical Health and Clinical Translation, School of Medicine, Barwon Health, Geelong, Australia; 9 Clinical Tuberculosis and Epidemiology Research Center, National Research Institute of Tuberculosis and Lung Diseases (NRITLD), Masih Daneshvari Hospital, Shahid Beheshti University of Medical Sciences, Tehran, Iran; 10 Nutritional Sciences Department, School of Nutritional Sciences and Food Technology, Kermanshah University of Medical Sciences, Kermanshah, Iran

**Keywords:** Food insecurity, Sleep, Meta-analysis, Systematic review

## Abstract

**Objective:**

We evaluated associations between food insecurity (FI) and the quality and quantity of sleep in adults (≥18 years).

**Design:**

The current study represented a systematic review and meta-analysis of observational studies.

**Setting:**

Databases of PubMed, Scopus, Embase and Web of Science were searched from inception until 6 June 2022. Meta-analyses were conducted using random-effects models, and effect sizes were reported as OR and 95 % CI.

**Participants:**

Data from ten eligible observational studies, including 83 764 participants, were included.

**Results:**

FI was associated with an increased risk of poor sleep quality (OR = 1·45; 95 % CI (1·24, 1·70), *I* ^2^ = 95, *P* < 0·001, *n* 7). Besides, subgroup analysis showed increased risk of poor sleep quality corresponding to the severity of FI across mild (OR = 1·31; 95 % CI (1·16, 1·48), *I* ^2^ = 0 %, *P* < 0·001, *n* 5), moderate (OR = 1·49; 95 % CI (1·32, 1·68), *I* ^2^ = 0 %, *P* < 0·001, *n* 5) and severe (OR = 1·89; 95 % CI (1·63, 2·20), *I* ^2^ = 0 %, *P* < 0·001, *n* 5) levels. Similarly, subgroup analysis by sleep problems showed that FI was associated with an increased the risk of trouble falling asleep (OR = 1·39; 95 % CI (1·05, 1·83), *I* ^2^ = 91 %, *P* = 0·002, *n* 3) and trouble staying asleep (OR = 1·91; 95 % CI (1·37, 2·67), *I* ^2^ = 89 %, *P* < 0·001, *n* 3). Moreover, FI was associated with the odds of shorter (OR = 1·14; 95 % CI (1·07, 1·21), *I* ^2^ = 0 %, *P* < 0·001, *n* 4) and longer sleep duration (OR = 1·14; 95 % CI (1·03, 1·26), *I* ^2^ = 0 %, *P* = 0·010, *n* 4).

**Conclusions:**

Collective evidence supports that FI is associated with poor sleep quality and quantity in adults. Preventative and management strategies that address FI may provide health benefits beyond improving nutritional status per se.

Food insecurity (FI) is characterised by the US Department of Agriculture as limited or uncertain access to nutritionally adequate and safe foods or the ability to acquire acceptable foods in socially acceptable ways due to limited financial resources^(1)^. FI represents a staggering global burden^(2)^. The prevalence of moderate or severe FI has substantially increased from 8·3 % in 2014 to 25·9 % in 2020, with a projected two billion individuals at risk of hunger^(3)^. The condition is associated with an increased risk of morbidity, including type 2 diabetes^(4)^, CVD^(5)^, anaemia^(6)^, metabolic syndrome^(7,8)^, stunting^(8)^, mental disorders^(9)^ and mortality^(10)^, highlighting the need for preventative and management strategies.

Sleep plays a significant role in the physical and mental health status^(11,12)^. Many factors are known to affect the quality and quantity of sleep, including ageing^(13)^, chronic diseases^(14)^, obesity^(15)^, occupational stress^(16)^, poor sleep environment^(17)^, smoking^(18)^, excessive caffeine^(19)^, alcohol^(20)^ and drinking consumption. Similarly, FI may contribute to impaired sleep behaviours^(21)^, albeit the evidence is less conclusive.

In general, available studies have reported positive associations between FI and poor sleep quality; however, results are less consistent on select sleep health outcomes, including sleep quality and duration^(22–27)^. To that end, Ding *et al*.^(22)^ and Jordan *et al*.^(25)^ have reported associations between FI and poor sleep quality across the mild to severe status. However, Grandner *et al*.^(28)^ showed only extreme levels of FI are related to poor sleep quality, unlike mild levels. Consistently, data on associations between FI and sleep duration are mixed. Troxel *et al*.^(29)^ reported that higher levels of FI were associated with a greater risk of short or long sleep duration compared with normal sleep duration (7–9 h). Similarly, Narcisse *et al*.^(30)^ and Jordan *et al*.^(25)^ showed that FI is associated with short sleep duration. In contrast, they reported a lack of association between FI and long sleep duration^(25,30)^. Conversely, Whinnery *et al*.^(31)^ exhibited FI is associated with both short and long sleep duration. Collectively, little can be concluded on the direction and magnitude of the relationship between FI and sleep behaviours.

To our knowledge, no meta-analysis has pooled evidence on the relationship between FI and sleep behaviours, despite a clear need. To address this knowledge gap, we conducted a systematic review and meta-analysis of observational studies to delineate associations between FI and the quality and quantity of sleep in adults (≥18 years). We also comprehensively evaluated factors that may influence these associations, including the severity of FI and biological and socio-demographic characteristics of study populations (e.g. age, sex, BMI, race, ethnicity, mental health, education and income status).

## Methods

### Systematic search and study selection

The work presented herein was carried out according to the Preferred Reporting Items for Systematic Reviews and Meta-Analyses (PRISMA)^(32)^. The study protocol was registered at the PROSPERO (Prospective Register of Systematic Reviews; registration identifier: CRD42021275645). A comprehensive literature search was conducted using the databases of PubMed, Scopus, Web of Science and Embase from inception until 6 June 2022. The search was performed using following medical subject heading (MeSH) and defined search terms without any language or date restrictions: ((‘Food Supply’[Mesh] OR ‘Food Supply’[Title/Abstract] OR ‘Food Supplies’[Title/Abstract] OR ‘FI’[Title/Abstract] OR ‘Food Insecurities’[Title/Abstract] OR ‘Food security’[Title/Abstract] OR ‘Food securities’[Title/Abstract]) AND (‘Sleep’[Mesh] OR ‘Sleep’[tiab] OR ‘insomnia’[tiab] OR ‘insomnias’[tiab] OR ‘sleep problems’[tiab] OR ‘sleep quality’[tiab] OR ‘sleep duration’[tiab] OR ‘sleep deprivation’[tiab] OR ‘sleep disturbance’[tiab] OR ‘sleep disorders’[tiab]) (see online Supplemental Table 1). Also, the references of retrieved records were evaluated manually to identify relevant citations for inclusion in our literature search.

### Eligibility criteria

Studies were included in the final analysis if they: (1) were observational (cross-sectional, cohort and case–control); (2) were conducted on adults (≥18 years); and (3) reported effect estimates in the form of OR, relative risk, or hazard ratio and with corresponding 95 % CI on associations between FI and sleep quality across short (≤6 h) or long (≥9 h) durations.

Exclusion criteria were studies: (1) that conducted on children or adolescents (<18 years); (2) that had insufficient data for inclusion in our analyses; and (3) that had with inappropriate designs, including interventional studies, reviews, letters, editorials, conference proceedings, notes or surveys.

### Study selection

The titles and abstracts of all identified records in our literature search were assessed independently by two authors (SM and M-AH-K), followed by a full-text review of eligible records. All discrepancies were resolved by consensus with a third investigator (HM).

### Data collection

Extracted data for each included study were as follows: (1) the first author’s name; (2) study publication year; (3) databases used; (4) study design; (5) study country of origin; (6) study sample size; (7) biological and socio-demographic characteristics of study populations (e.g. age, sex, BMI, race, ethnicity, mental health, education and income status where available); (8) level of FI severity; (9) assessment methods for FI and sleep behaviours; (10) relevant effect estimates; (11) study main findings; and (12) any adjusted analyses. Two investigators (SM and HM) independently extracted data for all included records using a standard information extraction template. Data extraction was reviewed by all other authors (S-NM, ST, MK, S-MG, S-PM, PA, SN-M and WM) for any potential extraction error.

### Quality assessment

Two investigators (SM and HM) independently examined the quality of each included study using the Newcastle–Ottawa scale^(33)^. The method of quality evaluation has been formerly described^(6,12)^.

### Statistical analysis

All statistical tests were conducted using STATA (version 14.0; Stata Corp.). To analyse associations between FI and sleep behaviours, fully adjusted risk estimates for poor sleep quality and short or long sleep duration were pooled. Pooled OR and 95 % CI were estimated using a weighted random-effects model per the DerSimonian–Laird approach^(34)^. The heterogeneity among the studies was examined by the Cochran Q and *I* ^*2*^ statistics (*I* ^*2*^ = (Q-df)/Q × 100 %; *I* ^*2*^ < 25 %, no heterogeneity; *I* ^*2*^ = 25–50 %, moderate heterogeneity; *I* ^*2*^ = 50–75 %, considerable heterogeneity, *I* ^*2*^ > 75 %, extreme heterogeneity). The heterogeneity was considered significant if the Q statistic had *P* < 0·1 or *I* ^2^ > 50 %. To identify the sources of heterogeneity, subgroup analyses were conducted based on FI levels (mild, moderate and severe)^(35)^, sleep problems (trouble falling asleep and difficulty staying asleep) and country (USA and Mexico). We also performed subgroup analyses based on age (<50 and ≥50 years), ethnicity/race (mixed and Latino) and number of participants (<4000 and >4000). Our subgroup analyses were justified based on eight recommended criteria of the Instrument to Evaluate the Credibility of Effect Modification Analyses (ICEMAN)^(36)^. We also performed meta-regression analyses to evaluate the link between the risk of sleep quality or quantity and heterogeneity between studies. Further, we performed sensitivity analyses by removing each study and recalculating the overall effect size to determine whether an individual study exerted undue influence. Funnel plots and results of Begg’s and Egger’s tests were used to assess publication bias. Results were considered significant at *P* < 0·05.

## Results

**Fig. 1:**
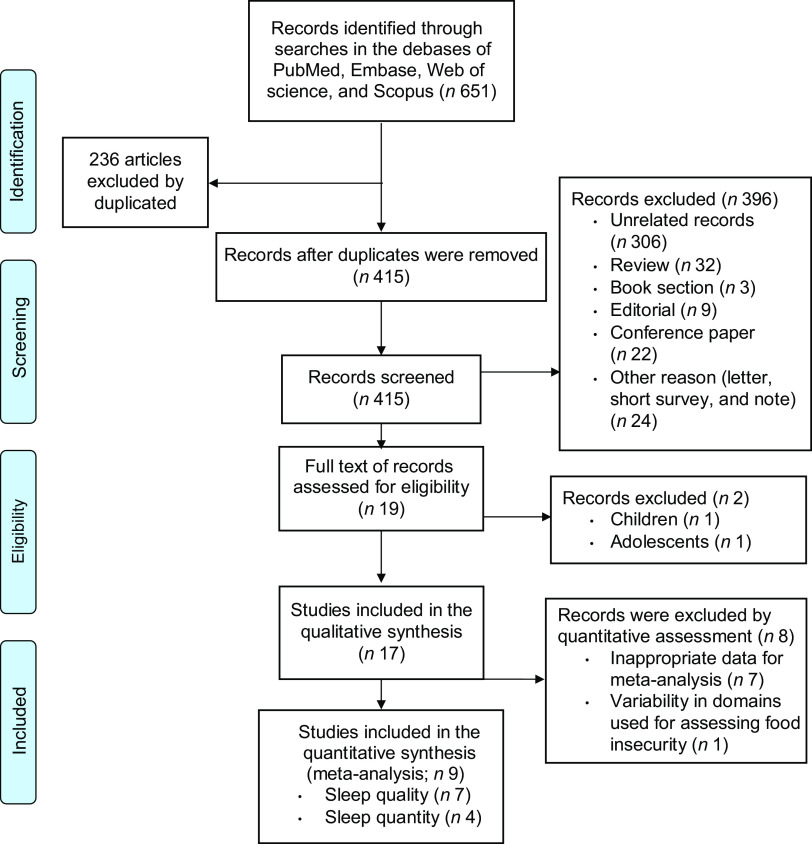
PRISMA flow diagram of the study

The systematic search resulted in 651 records (Fig. 1), of which 318 records were screened after removing duplicates. Of these 318 records, 302 were excluded because they did not meet our inclusion criteria, resulting in nineteen eligible studies for full-text evaluation^(22–31,37–42)^. Of these nineteen studies, ten were excluded because they were conducted on children^(40)^ or adolescents^(43)^, provided insufficient data for inclusion in our analyses^(37–39,41,42,44,45)^, or used variable domains to measure FI^(24)^. Together, nine eligible studies (*n* 83 764)^(22,23,25–31)^ were included in our study (Fig. 1).

All included studies had a cross-sectional design (Table 1) and were published between 2013 and 2021 and conducted in the USA^(22,23,26–31)^ and Mexico^(25)^. OR on the link between FI and quality and quantity of sleep were pooled across these nine studies for meta-analyses. Seven studies assessed poor sleep quality risk (*n* 47 439), and four reported sleep duration risk (*n* = 29 583).

**Table 1:** Summary of studies included in the meta-analysis

First author (year)	Database study design/country	Subjects: age (year) population size sex	Race/ethnicity	Level of food insecurity	Measure of food insecurity	Measure of sleep assessment	OR (95 % CI)	Main findings	Adjusted variables	Quality score
Grandner *et al*. (2013)	The 2007–2008 NHANES /Cross-sectional/USA	≥ 18 years *n* 4081 (51·95 % male and 48·05 % female)	Mixed	Household	USDA	Specific survey question	Sleep quality	Household food insecurity was associated with poor sleep quality	Age, sex, race/ethnicity, education, income, BMI, general health, physical health, immigration status and marital status	+7/10
Ding *et al*. (2014)	2005–2006, 2007–2008 and 2009–2010 cycles of NHANES/cross-sectional/USA	≥ 22 years *n* 15 961 (35·31 % male and 64·69 % female)	Mixed	Household	USDA	Specific survey question	Sleep quality	Household food insecurity was associated with poor sleep quality	Poverty income ratio, BMI, household size, race/ethnicity, education level, marital status, work schedule, poor mental health, general health condition, smoking status, alcohol consumption and menopause status	+8/10
Whinnery *et al.* (2014)	The 2007–2008 NHANES /cross-sectional/USA	≥ 18 years *n* 4850 (48·3% male and 51·6 % female)	Mixed	Household	USDA	Specific survey question	Sleep duration	Those with mild food insecurity were more likely to have short sleep durations.	Overall health, probable insomnia and probable sleep apnoea	+7/10
Jordan *et al*. (2016)	ENSANUT-2012/cross-sectional/Mexico	≥ 18 years *n* 11 356 (44·9% male and 55·1 % female)	Latina	Household	ELCSA	Specific survey question	Sleep duration and quality	Household food insecurity was associated with inadequate sleep duration and poor sleep quality among Mexican adults.	Age, sex, area of residence, household size, socioeconomic and BMI	+8/10
Narcisse *et al*. (2019)	Hawaiian and other Pacific Islanders (NHPI)/cross-sectional/USA	≥ 18 years *n* 12 619 (46·1% male and 53·9 % female)	Mixed	Household	Survey	Specific survey question	Sleep duration and quality	Food insecurity was associated with sleep quality and quantity	Age, sex, poverty level, education, employment and marital status	+8/10
Troxel *et al*. (2019)	Cross-sectional/USA	≥ 18 years *n* 785 (23·4% male and 76·4 % female)	Mixed	Household	USDA	Specific survey question	Sleep duration	Greater food insecurity was associated with a greater likelihood of short and long sleep	Age, sex, education, marital/cohabitation status, annual household income, presence of children in the home, use of Supplemental Nutrition Assistance Program (SNAP), length of residence in the neighbourhood, BMI and neighbourhood type	+8/10
Zein *et al*. (2019)	Cross-sectional/USA	≥ 18 years *n* 855 (31·2% male and 68·8 % female)	Mixed	Household	USDA	PSQI	Sleep quality	Food insecurity had significantly higher odds of poor sleep quality	Age, sex, race/ethnicity, place of residence, meal plan, employment, Pell grant status, university and parental education	+9/10
Nagata *et al*. (2019)	National Longitudinal Study of Adolescent to Adult Health/cross-sectional/ USA	≥ 18 years *n* 14 786 (51 % male and 49 % female)	Mixed	Household	USDA	Specific survey question	Sleep quality	Food insecurity was associated with poorer sleep outcomes	Age, sex, race/ethnicity, education, income, household size, BMI, smoking, alcohol and receipt of public assistance	+8/10
Hagedorn *et al.* (2021)	Cross-sectional/USA	≥ 18 years *n* 17 686 (25·1% male and 74·9 % female)	Mixed	Household	USDA	PSQI	Sleep quality	Food-insecure students had higher adjusted odds of having poor sleep quality	Age, sex, race, BMI, living on or off-campus, having dependents, having a disability, and having restless leg syndrome symptoms	+9/10

KNHANES, Korea National health and nutritional examination survey; ENSANUT-2012, The 2012 Mexican national health and nutrition survey; ELCSA, The Latin American and Caribbean food security scale; PSQI, Pittsburgh sleep quality index; IFLS5, Indonesian family life survey; PROMIS, patient-reported outcomes measurement information system; CES-D, epidemiologic studies – depression.

Results of the study quality assessment for each study are shown in Table 1. Briefly, quality assessments revealed that seven studies had high quality^(22,23,25–27,29,30)^ and two had medium quality^(28,31)^ (Table 1).

### Sleep quality

**Fig. 2:**
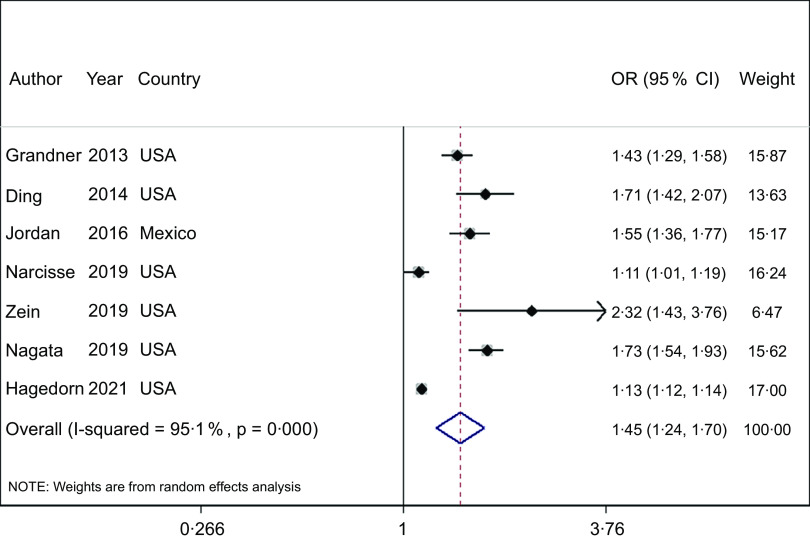
Forest plot showing the OR and 95 % CI of the association between food insecurity and the risk of poor sleep quality

FI was associated with an increased risk of poor sleep quality in adults (OR = 1·45; 95 % CI (1·24, 1·70), *P* < 0·001, *n* 7; Fig. 2). Studies were highly heterogenous (*I* ^*2*^ = 95 %, *P* < 0·001).

Subgroup analysis showed increased risk of poor sleep quality corresponding to the severity of FI across mild (OR = 1·31; 95 % CI (1·16, 1·48), *I* ^*2*^ = 0 %, *P* < 0·001, *n* 5), moderate (OR = 1·49; 95 % CI (1·32, 1·68), *I* ^*2*^ = 0 %, *P* < 0·001, *n* 5) and severe (OR = 1·89; 95 % CI (1·63, 2·20), *I* ^*2*^ = 0 %, *P* < 0·001, *n* 5) levels (Table 2). Similarly, subgroup analysis by sleep problems showed that FI was associated with an increased the risk of trouble falling asleep (OR = 1·39; 95 % CI (1·05, 1·83), *I* ^*2*^ = 91 %, *P* = 0·002, *n* 3) and trouble staying asleep (OR = 1·91; 95 % CI (1·37, 2·67), *I* ^*2*^ = 89 %, *P* < 0·001, *n* 3; Table 2). Also, subgroup analysis based on country revealed that FI was associated with an increased the risk of poor sleep quality across studies conducted within (OR = 1·44; 95 % CI (1·22, 1·70), *I* ^*2*^ = 95 %, *P* < 0·001, *n* 6) or out of (OR = 1·55; 95 % CI (1·36, 1·77), *n* 1) USA.

**Table 2:** Subgroup analysis to assess the associations between food insecurity and the quality and quantity of sleep

Subgroup	Number of studies	OR*	95 % CI	*P*-value	*I* ^2^ (%)	*P*-value for heterogeneity†	*P*-value for between subgroup comparisons‡
Poor sleep quality risk							
Food insecurity degree							
Mild	5	1·31	1·16, 1·48	<0·001	0·0	0·41	0·003
Moderate	5	1·49	1·32, 1·68	<0·001	0·0	0·50	
Severe	5	1·89	1·63, 2·20	<0·001	0·0	0·82	
Sleep problems							
Trouble falling asleep	3	1·39	1·05, 1·83	0·02	91·4	<0·001	0·258
Trouble staying asleep	3	1·91	1·37, 2·67	0·001	89·3	<0·001	
Country							
USA	6	1·44	1·22, 1·70	<0·001	95·1	<0·001	0·817
Mexico	1	1·55	1·36, 1·77	<0·001			
Age							0·525
< 50	6	1·41	1·20, 1·66	<0·001	95·2	<0·001	
≥50	1	1·71	1·42, 2·06	<0·001	–	–	
Race							
Mixed	6	1·44	1·22, 1·70		95·1	<0·001	0·817
Latina	1	1·55	1·36, 1·77		–	–	
Number of participants							
<4000	3	1·57	1·06, 2·31	0·02	95·5	0·023	0·738
>4000	4	1·42	1·16, 1·75	<0·001	95	0·001	
Adjustments							
BMI							
Yes	4	1·50	1·14, 1·97	0·003	96·8	<0·001	0·788
No	3	1·40	1·08, 1·81	0·011	90·5	<0·001	
Sex							
Yes	5	1·41	1·17, 1·70	<0·001	95·3	<0·001	0·697
No	2	1·54	1·29, 1·82	<0·001	62·7	0·101	
Mental health							
Yes	2	1·54	1·29, 1·82	<0·001	62·7	0·101	0·697
No	5	1·41	1·17, 1·70	<0·001	95·3	<0·001	
Education status							
Yes	4	1·60	1·17, 2·18	0·003	94·2	<0·001	0·448
No	3	1·35	1·09, 1·67	0·007	95·3	<0·001	
Income status							
Yes	3	1·48	1·06, 2·06	0·021	95·7	<0·001	0·955
No	4	1·44	1·16, 1·79	0·001	94·1	<0·001	
Short sleep duration risk							
Food insecurity degree							
Mild	2	1·03	0·86, 1·22	0·76	0·0	0·74	0·085
Moderate	2	1·01	0·84, 1·22	0·89	0·0	0·53	
Severe	2	1·59	1·16, 2·18	0·004	45·9	0·17	
Age							
< 50	2	1·21	1·08, 1·36	0·001	0·0	0·41	0·316
≥50	2	1·11	1·02, 1·19	0·01	0·0	0·85	
Race							
Mixed	3	1·14	1·05, 1·23	0·002	12·9	0·31	0·732
Latina	1	1·17	1·02, 1·35	0·028	–	–	
Number of participants							
<4000	2	1·17	1·02, 1·34	0·02	52·0	0·14	0·874
>4000	2	1·14	1·02, 1·27	0·02	0·0	0·53	
Long sleep duration risk							
Food insecurity degree							
Mild	2	1·02	0·83, 1·25	0·83	0·0	0·64	0·274
Moderate	2	1·05	0·82, 1·33	0·70	0·0	0·59	
Severe	2	1·31	0·98, 1·76	0·06	0·0	0·92	
Age							
< 50	2	1·13	0·99, 1·29	0·07	0·0	0·57	0·869
≥50	2	1·15	0·99, 1·34	0·07	2·6	0·31	
Race							
Mixed	3	1·16	1·02, 1·33	0·02	0·0	0·56	0·689
Latina	1	1·11	0·96, 1·29	0·16	–	–	
Number of participants							
<4000	2	1·20	1·04, 1·38	0·01	0·0	0·85	0·446
>4000	2	1·09	0·95, 1·25	0·22	0·0	0·49	

* Calculated by random-effects model.

† *P*-value for heterogeneity within the subgroup.

‡ *P*-value for heterogeneity between subgroups using meta-regression analyses.

To further explore the sources of heterogeneity, meta-regression analyses were conducted to identify any influence of FI degree, sleep problems, age, race/ethnicity, study sample size and adjusted risk estimates across different exposure categories (Table 3). Heterogeneity was decreased following meta-regression analyses based on FI levels (*P* = 0·003, *I* ^*2*^ = 0 %; Table 3 and see online Supplemental Fig. 1). However, sleep problems, age, race/ethnicity, number of participants, and studies that controlled for sex, BMI, mental, education, and income did not explain the sources of heterogeneity.

**Table 3:** Findings from meta-regressions

Poor sleep quality risk	Effect size, number of studies	OR	95 % CI	*I* ^*2*^ residual	*P*-value
Food insecurity degree	15	1·19	1·07, 1·32	0·0	0·003
Sleep problems	6	1·37	0·70, 2·69	90·47	0·258
Country	7	1·06	0·53, 2·14	95·1	0·817
Age	7	1·20	0·60, 2·39	95·22	0·525
Race	7	1·06	0·53,2·14	95·1	0·817
Number of participants	7	0·93	0·56, 1·55	95·24	0·738
Adjustments					
BMI	7	0·94	0·56, 1·57	95·66	0·788
Sex	7	1·09	0·63, 1·86	94·28	0·697
Mental health	7	0·91	0·53, 1·57	94·28	0·697
Education status	7	0·86	0·53, 1·37	94·66	0·448
Income status	7	0·98	0·59, 1·63	94·85	0·955
Short sleep duration risk					
Food insecurity degree	6	1·23	0·95, 1·59	37·82	0·085
Age	4	0·91	0·67, 1·22	0·0	0·316
Race	4	1·03	0·71, 1·49	12·89	0·732
Number of participants	4	0·98	0·69, 1·39	19·13	0·874
Long sleep duration risk					
Food insecurity degree	6	1·11	0·87, 1·43	0·0	0·274
Age	4	1·01	0·65, 1·57	0·0	0·869
Race	4	0·95	0·61, 1·47	0·0	0·689
Number of participants	4	0·90	0·58, 1·40	0·0	0·446

### Sleep quantity

**Fig. 3:**
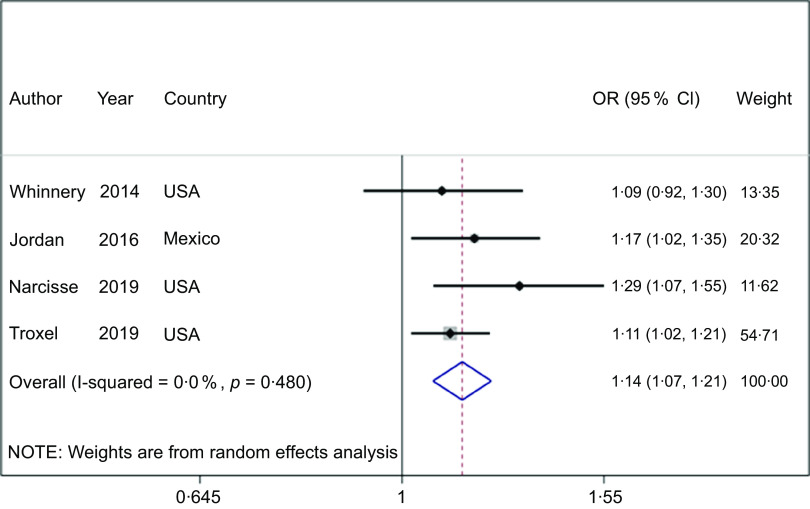
Forest plot showing the OR with 95 % CI of the association between food insecurity and the risk of short sleep duration

**Fig. 4:**
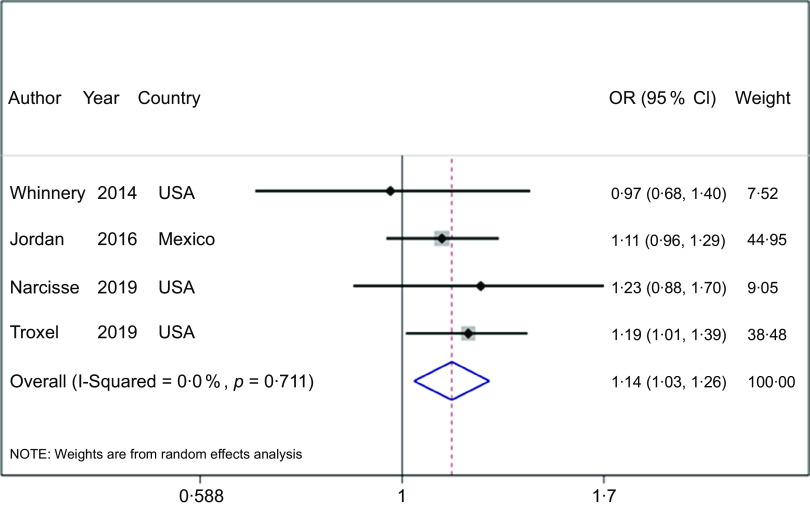
Forest plot showing OR with 95 % CI of the association between food insecurity and the risk of long sleep duration

FI was associated with an increased risk of short (OR = 1·14; 95 % CI (1·07, 1·21), *P* < 0·001, *n* 4) or long (OR = 1·14; 95 % CI (1·03, 1·26), *P* = 0·010, *n* 4) and sleep duration (Figs. 3 and 4, respectively), and studies were homogenous (All: *I* ^*2*^ = 0 %; All: *P* ≥ 0·05). In addition, subgroup analysis showed that a severe level of FI is associated with an increased risk of short sleep duration (OR = 1·59; 95 % CI (1·16, 2·18), *I* ^*2*^ = 46 %, *P* = 0·004, *n* 2; Table 2). In contrast, meta-regression analyses based on pooled FI levels, age, race and study size could not explain the sources of heterogeneity (All: *P* > 0·05, Table 3 and see online Supplemental Figs. 2 and 3).

### Sensitivity analysis and publication bias

**Fig. 5:**
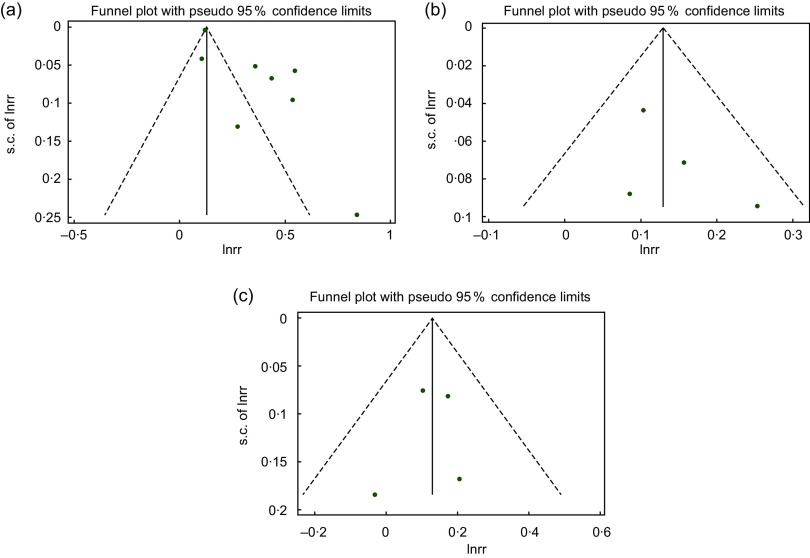
Funnel plot for evaluation publication bias in studies reporting OR and 95 % CI of the association between food insecurity and risk of poor sleep quality (a), short sleep duration (b), and long sleep duration (c)

Sensitivity analysis revealed that the pooled effect estimates were not affected by any single study included in our analyses. The Egger’s test (*P* = 0·01) revealed a publication bias for studies assessing the relationship between FI and the risk of poor sleep quality. However, the bias was not evident using Begg’s test results (*P* = 0·71) or a symmetric funnel plot (Fig. 5(a)). Further, we observed no publication bias in studies evaluating the link between FI and short (*P* = 0·30 for Begg’s test and *P* = 0·39 for Egger’s test; Fig. 5(b)) and long (*P* = 0·49 for Begg’s test and *P* = 0·73 for Egger’s test; Fig. 5(c)) sleep duration.

## Discussion

Few studies have examined the relationship between FI and non-nutritional health outcomes, including sleep behaviours. To our knowledge, the present work is the first to investigate associations between FI and the quality and quantity of sleep. The most significant finding of our study was that FI was associated with an increased risk of poor sleep quality in adults. Also, FI was associated with an increased risk of short and long sleep duration. Together, our findings highlight the adverse influence of FI on sleep behaviours.

Our observations add a novel dimension to current evidence about the negative influence of FI on sleep health status in the general adult population and extend previous reports. Our results are consistent with those of a cross-sectional study on patients with type 2 diabetes, and Bermúdez-Millán *et al.* ^(46)^ demonstrated that household FI is a common and potent household stressor related to suboptimal sleep quality through psychological distress. Similarly, Liu *et al.* corroborated associations between FI, frequent mental distress and insufficient sleep among adults across twelve states in the USA.^(39)^ Consistently, Pinto *et al.* ^(47)^ reported that FI is associated with increased odds (OR: 2·25; 95 % CI (1·11, 4·55)) of poor sleep quality in children.

The biological and psychosocial factors involved in mechanisms behind the association between FI status and adult sleep behaviours are less clear. However, this relationship may be mediated, at least partially, through mental health disorders (e.g. depression or depressive symptoms)^(48)^. FI is associated with an increased risk of depression^(49–51)^, anxiety^(52)^ or stress^(53,54)^. These mental health complications are known to be associated with adverse sleep quality^(55,56)^. Individuals affected by FI present with perceived powerlessness, disappointment, embarrassment and guilt, which may contribute to anxiety and depressive symptoms^(57)^. Furthermore, those with FI are more likely to consume convenience foods that are usually high in fat and refined sugars and are subsequently linked with poorer mental health through mechanisms explained in greater detail previously^(8,58,59)^. Stress and depression may also exacerbate FI status secondary hormonal imbalance, including aggravated cortisol secretion and dysregulation of the hypothalamic–pituitary–adrenal axis^(9)^. These alterations have been known to disrupt sleep^(60,61)^. Sleep disturbances can, in turn, alter appetite regulation medicated by increasing ghrelin and decreasing leptin levels. The compensatory mechanism of leptin reduces appetite and increases energy expenditure through the hypothalamic receptors^(62)^. Also, low leptin levels have been associated with poor sleep quality and a propensity for depressive symptoms^(62)^. Presently, the relative contributions of these individual factors to sleep behaviours are less conclusive by robust evidence, pointing to a research gap

This study has some strengths, including a comprehensive search strategy. This is the first meta-analysis to report associations between FI and sleep quality and quantity. Most studies included in our meta-analysis accounted for critical confounding factors. We performed several subgroup analyses to determine the source of the heterogeneity. However, important limitations should be acknowledged in the interpretation of our findings. Our work included cross-sectional studies. Therefore, no causality may be inferred on the link between FI and sleep behaviours. Most included studies relied on self-reported measures for FI and sleep. Accordingly, our observations are likely prone to over- or under-estimations of these measures secondary to the recall bias. Most (eight) studies were conducted in the USA; therefore, our findings may not be generalisable to low- and middle-income countries. Moreover, we observed considerable variability across studies in methods (e.g. surveys) used to measure FI and sleep outcomes, possibly contributing to measurement errors and a misclassification bias, which have been corroborated in systematic reviews and meta-analyses of this type^(63,64)^. Also, the assessment of FI and sleep behaviours occurred in different years, making it challenging to detect whether FI levels remained unchanged when sleep behaviours were evaluated. We observed significant heterogeneity among included studies for sleep quality. The heterogeneity was attenuated when the meta-analysis was subgrouped by the level of FI, and these results were approved following meta-regression analyses. However, other factors include sleep problems, country, age, race, number of participants and study adjustments did not explain the sources of heterogeneity. Our observations highlight the need for further research to elucidate the underlying factors and mechanisms that could explain the link between FI and poor sleep behaviours.

## Conclusions

The present meta-analysis of observational studies revealed that FI was associated with poor sleep quality and quantity in adults. Our observations extend the growing evidence on associations between FI and physical and mental health. Findings from the present work highlight the need for preventative and management strategies that address FI and sleep behaviours. Future well-designed longitudinal studies with larger sample sizes should confirm our observations.

## Supplementary material

For supplementary material accompanying this paper visit https://doi.org/10.1017/S1368980022002488. click here to view supplementary material
