# Supplementary material for: Food insecurity is associated with the sleep quality and quantity in adults: a systematic review and meta-analysis
Source: Public Health Nutr. 2022 Nov 23;26(4):792–802. doi: 10.1017/S1368980022002488 (PMC10131157; doi:10.1017/S1368980022002488)

| **Supplemental Table 1.** Search queries used across databases. | |
| --- | --- |
| **Database** | **Search query** |
| PubMed  (n=114) | ("Food Supply"[MeSH Terms] OR "Food Supply"[Title/Abstract] OR "Food Supplies"[Title/Abstract] OR "Food Insecurity"[Title/Abstract] OR "Food Insecurities"[Title/Abstract] OR "Food security"[Title/Abstract] OR "Food securities"[Title/Abstract]) AND ("Sleep"[MeSH Terms] OR "Sleep"[Title/Abstract] OR "insomnia"[Title/Abstract] OR "insomnias"[Title/Abstract] OR "sleep problems"[Title/Abstract] OR "sleep quality"[Title/Abstract] OR "sleep duration"[Title/Abstract] OR "sleep deprivation"[Title/Abstract] OR "sleep disturbance"[Title/Abstract] OR "sleep disorders"[Title/Abstract]) |
| Scopus  (n=217) | ( ( TITLE-ABS-KEY ( "Food Supply" ) OR TITLE-ABS-KEY ( "Food Supplies" ) OR TITLE-ABS-KEY ( "Food Insecurity" ) OR TITLE-ABS-KEY ( "Food Insecurities" ) OR TITLE-ABS-KEY ( "Food security" ) OR TITLE-ABS-KEY ( "Food securities" ) ) ) AND ( ( TITLE-ABS-KEY ( "Sleep" ) OR TITLE-ABS-KEY ( "insomnia" ) OR TITLE-ABS-KEY ( "insomnias" ) OR TITLE-ABS-KEY ( "sleep problems" ) OR TITLE-ABS-KEY ( "sleep quality" ) OR TITLE-ABS-KEY ( "sleep duration" ) OR TITLE-ABS-KEY ( "sleep deprivation" ) OR TITLE-ABS-KEY ( "sleep disturbance" ) OR TITLE-ABS-KEY ( "sleep disorders" ) ) ) |
| Embase  (n=156) | ( (("Food Supply" OR "Food Supplies" OR "Food Insecurity" OR "Food Insecurities" "Food security" OR "Food securities" )) AND ( ("Sleep" OR "insomnia" OR "insomnias" OR "sleep problems" OR "sleep quality" "sleep duration" OR "sleep deprivation" OR "sleep disturbance" OR "sleep disorders") )) |
| Web of Science  (n=164) | ((("Food Supply" (All Fields) or "Food Supplies" (All Fields) or "Food Insecurity" (All Fields) or "Food Insecurities" (All Fields) or "Food security" (All Fields) or "Food securities" (All Fields)) AND (("Sleep" (All Fields) or "insomnia" (All Fields) or "insomnias" (All Fields) or "sleep problems" (All Fields) or "sleep quality" (All Fields) or "sleep duration" (All Fields) or "sleep deprivation" (All Fields) or "sleep disturbance" (All Fields) or "sleep disorders" (All Fields))) |
| Search date: June 06, 2022 | |

**Supplemental Figure 1:** Meta-regression analyses for the risk of poor sleep quality, A) food insecurity degree, B) sleep problems, C) country, D) age, E) race, F) number of participants, G) body mass index, H) sex, I) mental health, J) education status, K) income status.


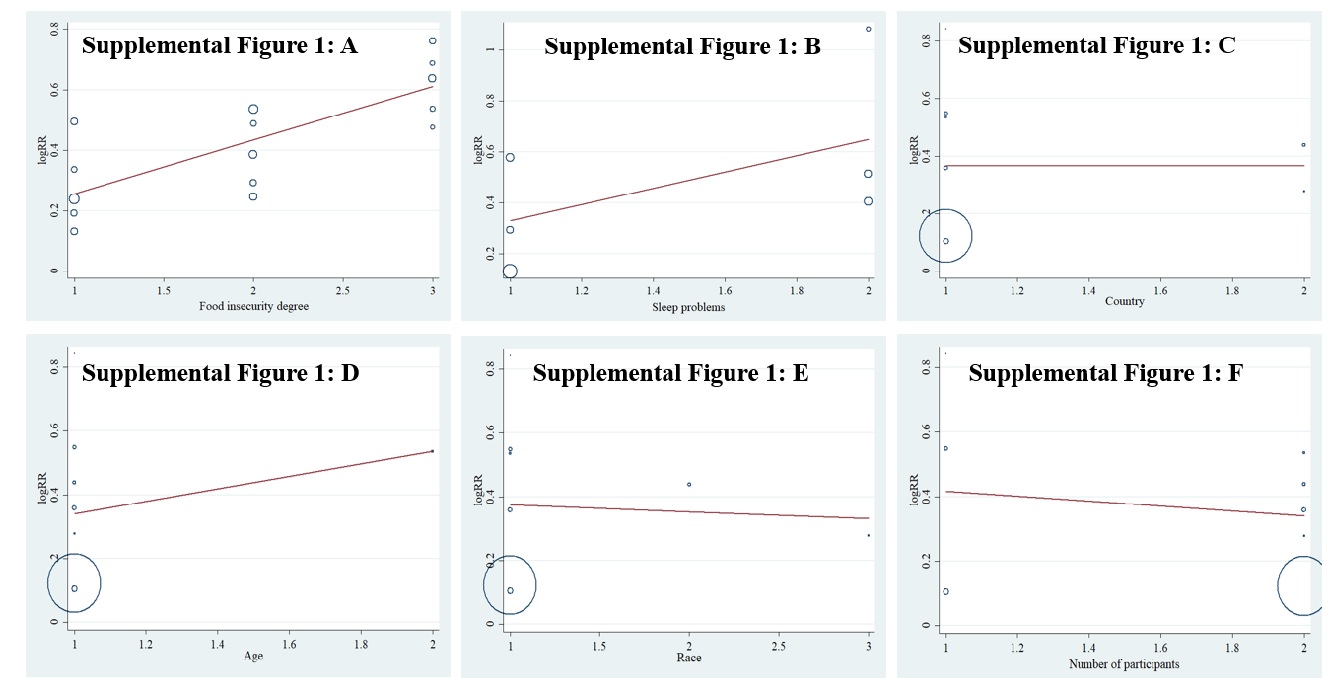


.


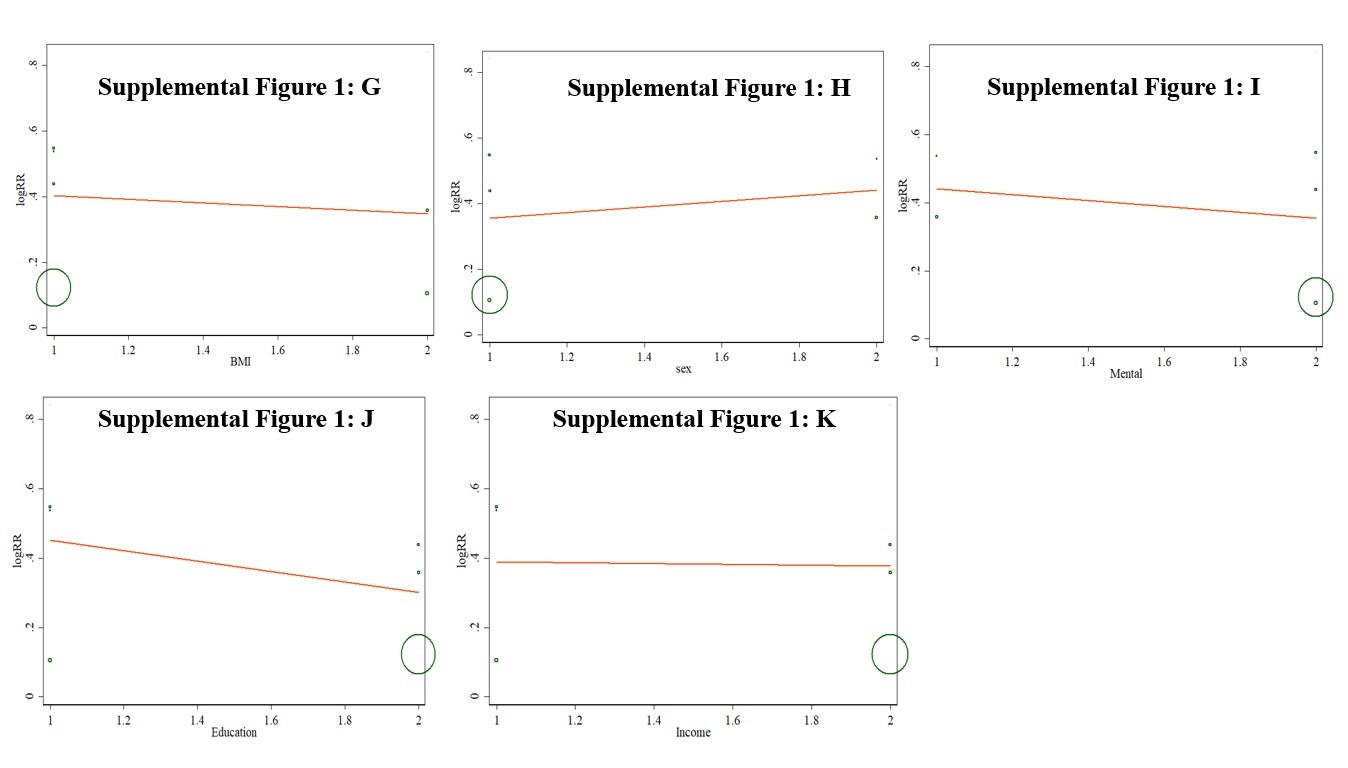


**Supplemental Figure 2:** Meta-regression analyses for short sleep duration risk, A) food insecurity degree, B) age, C) race, D) number of participants.


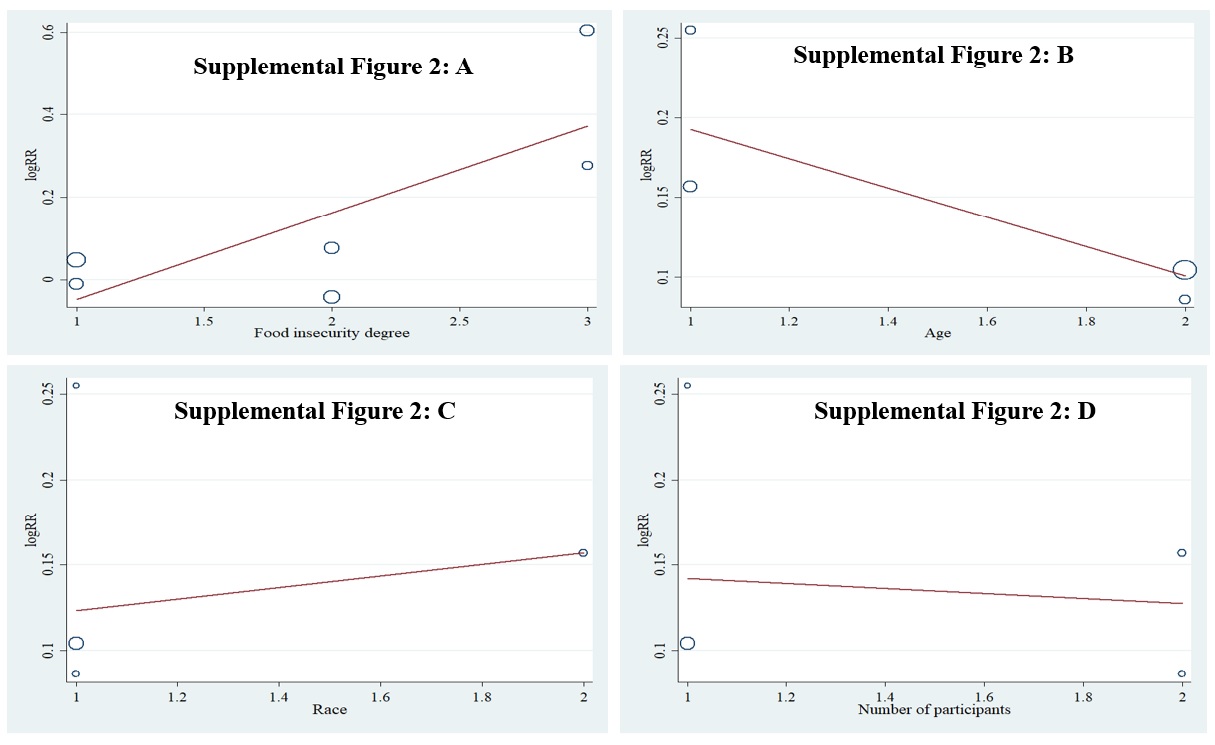


**Supplemental Figure 3:** Meta-regression analyses for long sleep duration risk, A) food insecurity degree, B) age, C) race, D) number of participants.


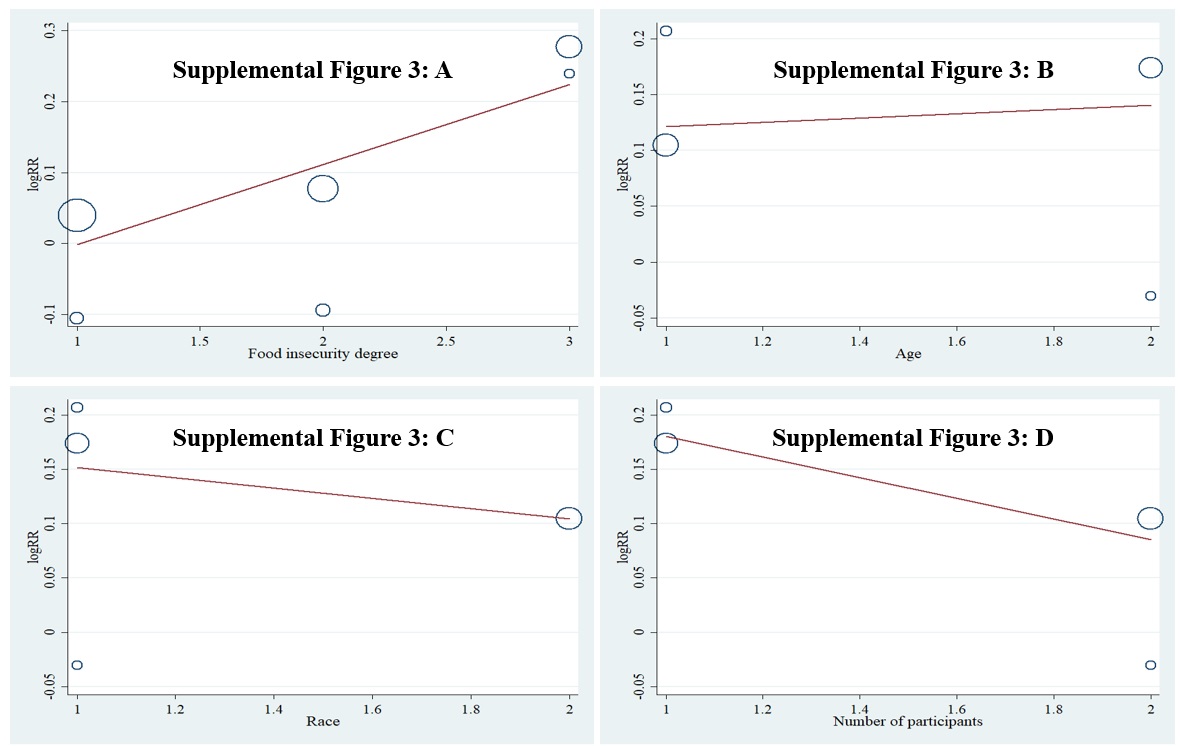

Supplement: Supplementary file 1 [file S1368980022002488sup001.docx]
